# Study relationship between the value of 17-OHP and the value of testosterone in monitoring for congenital adrenal hyperplasia

**DOI:** 10.1186/1687-9856-2013-S1-P124

**Published:** 2013-10-03

**Authors:** Ngo Thi Thu Huong, Nguyen Phu Dat, Nguyen Thi Hoan, Bui Phuong Thao, Nguyen Ngoc Khanh, Can Thi Bich Ngoc, Vu Chi Dung

**Affiliations:** 1 Pediatric Department, Hanoi Medical University,Vietnam; 2 National Hospital of Pediatrics, Hanoi, Vietnam

## 

Deficiency of 21-hydroxylase (21-OHD) is present in 90–95% cases of congenital adrenal hyperplasia (CAH), an autosomal recessive disorder. CAH affects severely on the physical development and reproductive function. In monitoring the disease and evaluating treatment outcome, presentation of salt wasting, electrolyte disturbance, androgenism, cushingoid features, plasma levels of 17-OHP, testosterone, Δ^4^-androstenedione, urinary levels of 17-OHCS, 17-CS, and bone age were used.

We aimed to study relationship between the value of the plasma 17–OHP and the value of the plasma testosterone levels to monitor treatment of CAH.

The study was prospective. We collected 82 CAH Vietnamese patients who were diagnosed of 21-OHD. The have been treated and monitored at the Vietnam National Hospital of Pediatrics. Hanoi, Vietnam in the period of 1/2007- 1/2010. The value of plasma levels of 17-OHP and testosterone were measured in combination with clinical symptoms every 6 months.

In 82 study’s CAH patients aged 1-15 years old, the group aged < 10 years old occupied 82.9%. The salt – wasting form occupied 75.6%; the simple virilizing form 24.4%. 59/82 of patients (71.1%) had successful treatment with the mean plasma level 17-OHP of 0.02-5.67 nmol/l and the mean levels of testosterone of 0.01- 8.02 nmol/l according to groups'ages. The mean levels of plasma 17-OHP and testosterone also increased with age and sex in the group of patients having failed treatment. It confirmed a positive relationship between the value of plasma 17 – OHP and levels of the testosterone with 95% significant confidence.

Each time of patient examination, besides clinical symptoms and plasma levels of 17-OHP should be done to evaluate treatment.

